# Barriers and opportunities to addressing social determinants of malaria in Wakiso District, Uganda

**DOI:** 10.1371/journal.pgph.0006922

**Published:** 2026-09-09

**Authors:** David Musoke, Edwinah Atusingwize, Allan Ssembuusi, Suzan Nakalawa, Kevin Deane

**Affiliations:** 1 Department of Disease Control and Environmental Health, Makerere University School of Public Health, Kampala, Uganda; 2 Faculty of Arts and Social Sciences, School of Social Sciences and Global Studies, The Open University, Milton Keynes, United Kingdom; University of Oxford, UNITED KINGDOM OF GREAT BRITAIN AND NORTHERN IRELAND

## Abstract

Malaria remains a significant public health threat in sub-Saharan Africa, particularly in Uganda, where it is the leading cause of morbidity and mortality. There is limited evidence on factors related to social determinants within ongoing malaria control efforts in the country. This study explored barriers and opportunities in addressing the social determinants of malaria in Uganda. A qualitative study was conducted in Wakiso district. Data were collected through 10 focus group discussions with community members and community health workers (CHWs), 11 in-depth interviews with heads of households, and 14 key informant interviews with local leaders, health officials, and non-governmental organisation (NGO) staff. Participants were purposively selected based on their knowledge and experience regarding malaria. Data were thematically analyzed using Atlas.ti. (version 6) and Nvivo (version 14). The study identified three key barriers and two opportunities to addressing the social determinants of malaria. The barriers were: 1. a curative-focused approach in national policies and private healthcare highlighting minimal commitment in addressing these determinants; 2. inadequate coordination among government institutions and partner organisations in addressing social determinants of malaria; and 3. complacency at both community and government levels due to malaria’s endemic nature. The opportunities identified were: 1. the presence of multiple stakeholders actively involved in malaria control including the Ministry of Health and other entities such as NGOs; and 2. the existence of robust government structures at national, district, and community levels including CHWs, health inspectors, malaria focal persons and the model homes initiative which provide a strong foundation to address social determinants of malaria in the country. Despite opportunities in addressing the social determinants of malaria, several barriers were identified. Overcoming these barriers requires a shift towards integrated public health strategies that advocate prevention, foster robust coordination, and leverage stakeholder engagement including communities to contribute to malaria elimination.

## Introduction

Malaria remains a major public health threat in tropical regions, particularly in sub-Saharan Africa which faces a disproportionate burden [1,2]. Despite being both preventable and treatable, the disease continues to inflict immense suffering and economic strain in endemic countries such as Uganda [2]. Historically, malaria has been a persistent health challenge, but concerted global efforts have demonstrated that elimination is achievable, as evidenced by recent successes in countries such as Egypt [3], Cape Verde [4], and El Salvador [5]. These achievements highlight the potential for progress when comprehensive and sustained strategies are effectively implemented in affected countries. Despite this success, many malaria endemic countries continue to face severe morbidity and mortality particularly among high-risk groups such as children [2].

The path to global malaria eradication is still faced with numerous challenges. According to the recent World Health Organization (WHO) 2025 malaria report, an alarming estimated 610,000 malaria-related deaths occurred in 2024, with the African region accounting for an overwhelming 95% of global malaria cases that year [6]. Over half of these deaths occur in sub-Saharan African countries, including Uganda contributing 4.7% in the third position after Nigeria and the Democratic Republic of the Congo with 24.3% and 12.5% respectively [6]. The impact of malaria on Uganda’s health system and household economies is profound. Indeed, the disease remains a leading cause of outpatient visits, accounting for 40% of such consultations, and contributing significantly to hospital admissions (25%) and overall mortality (14%) in the country [7]. This persistent malaria burden places immense pressure on an already strained healthcare system and severely impacts household productivity and economic stability, particularly in vulnerable rural and underserved communities where recurrent disease upsurges continue to hinder progress. With only four years remaining to achieve the health-related Sustainable Development Goals (SDGs), the ambitious global technical strategy for Malaria (2016–2030) aims to reduce cases and deaths by at least 90% compared to 2015 levels [8]. However, advancement towards these critical targets continues to lag behind expectations. For instance, there was only 3% decrease in malaria case incidence in 2020 compared to 2015, with cases having increased from 218 to 229 million [8]. This therefore necessitates an urgent re-evaluation and intensification of efforts and approaches to combat the disease effectively.

Controlling malaria in high-burden settings such as Uganda requires a paradigm shift towards a more holistic and comprehensive understanding of its underlying social determinants. These factors play a crucial but often underestimated role in shaping disease transmission patterns, influencing individual and community vulnerability, and ultimately determining health outcomes [9–11]. Beyond direct exposure to mosquitoes, factors such as poverty, inadequate and unsafe housing conditions, as well as limited access to timely and quality healthcare services greatly influence individuals’ susceptibility to malaria infection and their capacity to seek appropriate treatment [11,12]. In addition, poor environmental sanitation, inconsistent and improper use of protective measures such as insecticide-treated nets, and occupational exposures, particularly in agricultural and mining areas, further exacerbate the risk, intensifying the challenge in low resource regions [12–14]. Unfortunately, although the social determinants of malaria are increasingly recognized, Uganda’s national malaria control policy gives limited attention to these broader factors [15]. The policy primarily highlights poverty as a contributing factor, while other critical determinants such as structural interventions including housing quality, urban planning, multisectoral action, employment conditions, and broader environmental exposures remain largely overlooked in policy design and implementation. A similar lack of clear efforts to address social determinants is noted in the Uganda Malaria Elimination Strategic Plan 2021/2025 and 2026/2031 [16]. This is concerning due to the rapid urbanisation in the country as well as introduction of new cities and other structures that come with new challenges such as congestion, limited accommodation and high unemployment [17]. With the current evidence, addressing these social determinants should be essential for developing targeted, equitable, and sustainable interventions that move beyond purely biomedical approaches [13,18]. Failure to address these deep-seated social drivers will render efforts to control and eliminate the disease insufficient. This would eventually lead to persistent malaria transmission, high preventable deaths, and a widening of health inequities, especially among the most vulnerable populations [9].

Despite growing recognition of the role social determinants play in shaping malaria outcomes, there remains limited evidence on the specific challenges and opportunities involved in addressing these factors [18]. Existing studies have highlighted how poverty, poor housing, limited education, and inadequate access to health services contribute to persistent malaria transmission [19,20]. However, there is still a gap in understanding how these factors intersect in practice in endemic countries. Crucially, there is also limited literature on how various stakeholders from community members to national policymakers perceive, prioritize, and respond to these social determinants within the context of ongoing malaria control efforts. Understanding these perceptions is vital as it directly impacts the design and implementation of context-specific interventions. Indeed, early engagement of stakeholders and understanding their perceptions regarding malaria has been found to be critical in successful implementation of malaria control strategies [21]. This study therefore addressed this critical knowledge gap by undertaking an in-depth exploration of the barriers and opportunities associated with addressing the social determinants of malaria in Uganda. By engaging key stakeholders, this research sought to contribute to understanding the root causes of vulnerability to malaria. Additionally, the study explored the extent to which the social determinants of malaria as a policy agenda can be engaged with to inform comprehensive, equitable, and sustainable strategies for malaria control, as well contributing towards elimination by 2030.

## Methodology

### Ethics statement

The study was approved by Makerere University School of Public Health Research and Ethics Committee (SPH-2024-578) and registered at the Uganda National Council for Science and Technology (HS4271ES). The study also received a favourable opinion from the Open University Human Research Ethics Committee (2024-0503-2). Administrative permission was obtained from the district and local authorities in areas where the study was conducted before its commencement. RAs were guided by field mobilizers who introduced them to the community and helped in building rapport. All participants were aged 18 years and above and provided written informed consent which was obtained in the most appropriate language understood. Informed consent was also obtained for audio recording of all the discussions. Participants were informed about their voluntary participation, freedom to terminate their participation at any time without any penalty, and liberty of not answering any questions they were not comfortable with. Codes were used for anonymity and no participants’ names and contact information were recorded anywhere during the study.

### Study area and design

The study was conducted in Wakiso district in central Uganda, specifically Busiro South Health Sub District (HSD). Wakiso district has an estimated population of 3,411,177 according to the recent 2024 national census [22]. Literature shows that Wakiso is among the districts in Uganda where malaria is endemic [23]. The district has a total of seven HSDs including Entebbe Municipality, Busiro North, Busiro South, Busiro East, Kyaddondo North, Kyaddondo South and Kira Municipality. Busiro South was purposely selected for its involvement in the study due to earlier community health work done in the area by the research team. The HSD has an estimated population of 390,754 people living across three Town Councils (Kajjansi, Kasanje, and Katabi) and one Sub County (Bussi) [23]. Busiro South has one health centre IV (Kajjansi), four health centre IIIs (Nakawuka, Kasanje, Zzinga, and Bussi) and three health centre IIs (Nsaggu, Nalugala, and Kitala). Health centre IIs, IIIs, and IVs are primary care facilities in the Ugandan context that provide services to majority of the population throughout the country. This was a cross-sectional study that used qualitative data collection methods of key informant interviews (KIIs), focus group discussions (FGDs), and in-depth interviews (IDIs).

### Sampling

Sampling of study participants was purposive based on individuals’ knowledge, expertise and experience regarding malaria by ensuring that they were either people living in the study community for at least six months, or officials engaged with the malaria programme in the district. A total of 10 FGDs were conducted, each composing 6–8 community members who were identified to ensure a comprehensive mix and representation across their community. The FGDs were conducted with community members and community health workers (CHWs) to assess their perceptions and experiences of social determinants of malaria, including the related barriers and opportunities. Community members were included in the FGDs regardless of whether they had recently suffered from malaria or not to solicit for their general perceptions. The FGDs were organized with gender and age considerations, having separate ones for males and females, and adults (40 years and above) and youth (18–39 years). In addition, 11 IDIs were conducted to explore lived experiences regarding barriers of addressing social determinants of malaria during control of the disease. These IDIs were conducted among selected community members, especially heads of households (both male and female) who had been recently significantly affected by malaria (individually or their families) regarding morbidity and mortality. The IDI participants were selected in a way that ensured a representation of households that had experiences of losing a member to malaria, as well as those that had other malaria related experiences. In addition to FGDs and IDIs, 14 KIIs were conducted as part of the study. These key informants were purposively selected based on their roles and related involvement and knowledge of malaria prevention programmes in the district. The KIIs represented a wide range of roles and settings, including officials from the Ministry of Health (MOH), district level leaders and health officials, researchers from universities, social workers, health inspectors, private and public health facility managers including drug shop owners, project managers from implementing partners, religious leaders from both Islam and Christian denominations, as well as local council leaders in rural and urban villages. The total number of KIIs, IDIs and FGDs who participated in the study was informed by saturation levels during data collection (field work was ended when no new information emerged through any additional interviews and discussions). All study participants were approached with the support and guidance of local leaders and CHWs in the study area which helped to ensure that they were appropriate for the research.

### Data collection

Data was collected between 7^th^ June to 18^th^ August 2024 by qualified research assistants (RAs) with knowledge and experience in conducting qualitative research, in addition to having a minimum of a bachelor’s degree. RAs were also trained on the study objectives and research ethics by the researchers (DM - male and EA - female). The training included re-orientating RAs on the social determinants of health using the Dahlgren-Whitehead model [24]. The KII, FGD and IDI guides, informed by all layers of the Dahlgren-Whitehead model, were developed by the research team and used to collect data among the respective selected participants. The use of this conceptual framework played a crucial role in helping study participants understand what was meant by the social determinants of health, given that this term would have been unfamiliar to many. Similar questions were asked for all the three tools that focused on understanding of the social determinants of malaria, malaria prevention efforts, as well as facilitators and barriers to the social determinants approach. In addition, the IDI participants were asked an extra question on how malaria affected them and their family in recent years. KII participants were also asked about any existent guidelines or policies regarding malaria prevention and control and how they addressed social determinants of malaria. This included how easy it was to bring discussions on social determinants into the policy making process for malaria control. The tools were piloted in one of the sub-counties in Wakiso district which was not involved in the study to assess suitability of the tools and re-orient teams on actual field expectations. The tools focused on collecting participants’ general views about malaria, their interpretations and real-life examples of the social factors that shape malaria, how malaria prevention efforts were being implemented in their communities, and the challenges to addressing these social determinants. *Luganda,* the most spoken local language in the area, was used for FGDs and IDIs. However, KIIs were conducted in either English or *Luganda* depending on the preference of participants. The FGDs were conducted at communal places in the community such as churches, schools, and community halls while IDIs were held at the households of the selected participants. The KIIs were held at an appropriate place as guided by the key informants including their homes or workplaces. The IDIs, FGDs and KIIs together offered an opportunity to gain an understanding of the barriers and opportunities of addressing the social determinants of malaria through their experiences. The research team ensured that privacy was maintained during collection of data across the various methods. The average duration of the various data collection methods was 30–60 minutes (for the KIIs and IDIs), and 1.5 to 2 hours (for the FGDs). In addition to note taking, all the interviews and discussions were audio recorded. All individuals that were approached accepted to take part in the study.

### Data analysis

All audio recordings were transcribed verbatim by the RAs and thematic analysis principles [25] were used. The transcripts in *Luganda* were translated to English by the RAs who were proficient in both languages before analysis. The analysis involved the researchers reading the transcripts several times to familiarize themselves with the data. This was followed by two rounds of coding, independently conducted by EA and KD using a coding framework informed by the Dahlgren and Whitehead conceptual map of social determinants of health. Coding was conducted with the support of Atlas.ti. (version 6) and Nvivo (version 14). Two software packages were used for the analysis because the process was carried out by two independent researchers (EA and KD), with each one using the software predominantly used at their institution. EA, KD and DM discussed and agreed on the final codes. Codes were compared, and grouped to form sub-themes, and then themes inductively. The grouping of codes and theme development was an iterative process with consensus of all the three researchers. Consensus meetings between the researchers primarily involved discussions about labels and interpretation of codes and themes, giving justification for each and exploring alternative labels in view of study objectives. A shared codebook that was developed by the researchers guided the entire analysis process. Overall, coding reliability was ensured through iterative consensus meetings where the two researchers met to compare coding, discuss discrepancies, and refine codes. The themes generated from the analysis were presented at a stakeholder workshop with 30 participants including selected study participants, Ministry of Health representatives, and other stakeholders.

### Reflexivity statement

The team comprised of researchers from Uganda and the UK with a background in various fields including environmental health, public health, social sciences, and development economics. The Ugandan team members (DM, EA, SN, and AS) had experience in malaria both as researchers, as well as locals who had encountered the disease. Whereas some of the Ugandan researchers had worked in the study area before, they were not involved in field work activities including stakeholder engagement and selection of participants. Indeed, the RAs, who had vast experience in research including qualitative methods, led the field engagements including data collection. The primary Ugandan researchers (EA and DM) took lead in the study activities in-country, with support of the UK partner (KD). Furthermore, we reflected on potential power dynamics between the university-based researchers and rural community members. Specifically, right from designing the study, we made considerations to ensure honest reporting during FGDs and IDIs first of all by building trust with participants through directly working with community health workers and leaders to mobilise them. Secondly, the consenting process was intentionally elaborate on ethical issues such as voluntary participation and data confidentiality. The research assistants’ experience in qualitative research also allowed for in-depth engagement with participants, including probing and presentation skills, that create relaxed environments, although such power dynamics can be exclusively eliminated. Data analysis and interpretation of results involved researchers from both Uganda and the UK, with several meetings held both in-person and virtually. The workshop that was held involving many of the participants ensured that they had an opportunity to contribute to the discussions and confirm the final findings that emerged from the study.

## Results

From the 100 participants that took part in the KIIs, FGDs and IDIs (Table 1), three barriers and two opportunities to addressing the social determinants of malaria emerged. The barriers to addressing the social determinants of malaria were curative focused interventions, inadequate coordination, and complacency. The opportunities to addressing the social determinants of malaria were multiple stakeholders, and existing government structures.

**Table 1:** Demographic characteristics of the participants.

	Sex		Age (years)				Education				Occupation			
Group	Male	Female	18-29	30-39	40-49	≥ 50	No formal	Primary	Secondary	Tertiary	Employed	Self-employed	Farmer	Student
IDIs	5	6	0	2	7	2	0	4	6	1	0	7	4	0
KIIs	9	5	0	2	11	1	0	0	4	10	8	6	0	0
**FGDs**														
Mixed rural	4	4	1	1	6	0	1	4	2	1	0	5	3	0
Mixed urban	3	3	2	1	0	3	0	0	5	1	3	3	0	0
Female rural	N/A	8	2	2	2	2	0	7	1	0	0	3	5	0
Female urban	N/A	8	2	2	2	2	1	1	4	2	1	7	0	0
Male rural	8	N/A	0	2	4	2	0	3	4	1	0	6	2	0
Male urban	6	N/A	0	0	1	5	0	0	4	2	1	3	2	0
Youth rural	3	3	3	3	N/A	N/A	0	2	4	0	1	5	0	0
Youth urban	5	3	4	4	N/A	N/A	0	0	5	3	1	6	0	1
CHW rural	3	6	1	3	0	5	0	5	3	1	1	1	7	0
CHW urban	2	6	1	1	5	1	0	1	3	4	0	1	7	0
Total (FGDs)	34	41	16	19	20	20	2	23	35	15	8	40	26	1
**Total** **(FGDs, IDIs, KIIs) = 100**	**48**	**52**	**16**	**23**	**38**	**23**	**2**	**27**	**45**	**26**	**16**	**53**	**30**	**1**

* IDI – in-depth interview; KII – key informant interview; FGD – focus group discussion; CHW – community health worker.

### Barriers to addressing social determinants of malaria

#### Curative focused interventions.

Findings indicated that the national malaria control policies and related government interventions prioritized treatment over preventive measures including addressing the social determinants. Some participants felt that the government’s minimal commitment to addressing the social determinants of malaria was the main reason why the disease was persistent in the country. However, other participants attributed it to delays and inconsistencies in preventive interventions such as the mass distribution of mosquito nets by the government, as it prioritized medical supplies. Some key informants indicated that health workers focused on educating patients within the facilities rather than sensitizing the healthy ones in communities on preventive measures.

*“You cannot talk about prevention when you are waiting for people to come to the health facility. Government funding to me is put on the treatment aspects and that is why you realize that the government is doing a lot of construction of the health facilities, recruiting so many health workers and delivering medicines.”* [KII, Wakiso district official]

Additionally, some participants were concerned that malaria management was treated as a business by private health facilities at the expense of targeting the social factors that contribute to the occurrence of the disease. Profit driven practices led to overcharging patients, incorrect prescription of doses, as well as financial interests over effective treatment. It was noted that vulnerable populations, especially the poor, often received sub optimal care due to their inability to afford full treatment. It was also reported that some patients had a culture of doing self-treatment for malaria. For instance, there was a reported increasing habit of people buying drugs over the counter when they suspected having malaria and by the time they sought rightful medical attention, the disease had worsened.

*“These health workers have made malaria treatment a business, because the moment you arrive at the health facility, you are asked for money even before investigations are completed. It is real business as if they are happy that people are getting sick of malaria.”* [Respondent 2, FGD men urban]

#### Inadequate coordination.

Lack of adequate collaboration and coordination between government institutions and partner organisations in malaria control were reported. Key informants noted that MOH, town councils and other relevant agencies had failed to effectively coordinate interventions related to social determinants of malaria and prevention strategies, resulting in fragmented efforts. For instance, they believed that the majority of breeding places for mosquitoes were manmade for example resulting from government activities such as road construction. However, the stakeholders responsible for addressing such problems were not working together to eliminate them. Participants thought that the Ministry of Works and Transport, responsible for constructing roads, should fill the open places left after construction that may act as breeding sites for mosquitoes. Participants also raised concerns over operations that left temporary and disjointed solutions. Some key informants felt that the few NGOs that focused on addressing the social determinants of malaria and the disease in general were competing and working in silos instead of operating together towards malaria control.

*“You know in the NGO business, all of us are looking at how best we can shine, but I think one thing that we need to appreciate is that it’s not about you shining, it’s about the community that you are in. So, I think partners should accept to work together. We have talked about this for a long time but I have never seen it happen. So, when are we going to integrate? I think we just need to work around this issue.”* [KII, NGO staff]

#### Complacency.

Participants mentioned complacency and laxity at both community and government levels as one of the barriers to addressing malaria and its social determinants. Most participants attributed this laxity to the endemic nature of the disease which people had got used to and believed that nothing much could be done about it. In fact, some participants reported that some people perceived malaria as a less serious health threat compared to other diseases such as COVID-19 to the extent that a malaria death was regarded as a usual one. Many participants also believed that the government was not doing enough since the efforts and investments to address the social determinants of malaria were not comparable to what was done during response to the COVID-19 pandemic.

*“Generally, being an endemic disease, I think there is laxity of people, hence getting used to it. You find that there is low risk perception that even a death of malaria is regarded as a normal death. And when you compare it to these other deaths like for COVID, one death could scare the entire country. But a malaria death, it is normal, people go and burry and get back to their businesses.”* [KII, NGO staff]

MOH was also noted as being reluctant and not doing adequate follow-up of their malaria programmes in communities. In addition, the public health department of local authorities was perceived as negligent of the social determinants of malaria, thereby slowing efforts of preventing the disease. Some participants therefore believed that the non-use of mosquito nets, laziness in keeping homes and communities clean, and reluctancy in seeking the rightful malaria treatment were resulting from such laxity and complacency. Participants further noted that government’s complacency was observed in the allocation of limited funds to MOH hence not sufficient to support the various planned activities, including addressing the social determinants of malaria. This left the ministry to rely on external funding which many times has its own priorities.

*“When you listen to the recent national budget reading, some ministries were given enough money. But when you listen to the amount of money that was given to the Ministry of Health, you will know that it wasn’t enough. Even if you try to fight this malaria, you may find that you will not have enough funds to do so.”* [Respondent 5, FGD males rural]

In addition, some CHWs who were involved in treating sick children suffering from malaria were reluctant in carrying out this role, claiming that the disease was no longer a problem. Such CHWs said that their main concerns were other diseases such as diarrhoea and pneumonia. Complacency was also believed to be the reason why some parents no longer had time to take their children suffering from malaria for treatment but rather sent them with their young siblings to take care of them when the parents were at work. It was also reported that community members, especially the educated, were no longer interested in community health talks and meetings where they used to learn about health issues such as malaria and its social determinants.

*“I was just in a discussion and we were hearing a report from a community health worker and she was saying, malaria is no longer a problem, everyone sleeps under a mosquito net. She said that the key problem is diarrhoea and pneumonia, hence wondered why we were still giving her more packs of malaria rapid diagnostic tests”.* [KII, NGO staff]

### Opportunities for addressing social determinants of malaria

#### Multiple stakeholders.

The presence of multiple stakeholders including NGOs, local leaders, and policy makers was mentioned to provide an opportunity to strengthen initiatives for addressing social determinants of malaria, as well as broader disease control efforts. It was noted that several NGOs and health partners supported malaria prevention through health education, community engagement, and resource mobilization. Some of the mentioned partners included MOH, Makerere University School of Public Health, BRAC Uganda, Action for Rural Women’s Empowerment, Buganda Kingdom, Malaria Consortium, Living Goods, Uganda Virus Research Institute, and WaterAid. These stakeholders were appreciated for working with communities in preventing and addressing malaria and its social determinants, especially in children under five years and pregnant mothers. For instance, the stakeholders were seen to be instrumental in enhancing the capacity of CHWs who did sensitisation in communities, as well as training them on how to test for malaria using rapid diagnostic tests as part of the integrated Community Case Management (iCCM) programme.

*“There are really many NGOs supporting malaria prevention and control. There are several roles they play in the community. Going ahead, if you look at their thematic areas, most of them are into hygiene and sanitation, they do a lot of education, sensitization and work around communities, working with the local governments.”* [KII, Wakiso district official]

In addition, it was noted that the government had established partnerships with various local and international organisations such as the Global Fund and Bill and Melinda Gates Foundation to fund initiatives directed towards addressing social determinants of malaria. These collaborations were reported to strengthen efforts to prevent malaria, as well as expanding outreaches to vulnerable and at-risk populations, such as people with disabilities and children under five years respectively.

*“So, we are seeing more and more funding coming in, we are seeing external partners picking a lot of interest to support malaria control. Global Fund, President’s Malaria Initiative, Bill and Melinda Gates, even national funding. Even the government is putting in some resources to control the disease.”* [KII, Researcher]

Furthermore, participants mentioned that stakeholders such as local leaders and community members were interested, open to new ideas, and yearned for more evidence regarding strategies for addressing the social determinants of malaria. This was seen as something that would motivate the uptake of these strategies in communities if they were to be implemented.

*“I think for me, we need to appreciate that the social determinants actually impact malaria. So, as we develop, be it preventive, or curative services, some of these aspects should also be looked into. What is core here is that we don’t have information on what extent the social determinants contribute to controlling the disease hence we are eager to learn.”* [KII, local council leader]

#### Existing government structures.

Participants indicated that the existing structures at national, district and community levels including health inspectors, malaria focal persons, CHWs, and model homes in some communities were a great opportunity to explore in addressing the social determinants of malaria. MOH and local government structures were also commended to have provided a strong foundation to address the social determinants of malaria in the country. Participants reported that government efforts were also supported by national malaria control policies such as the Public Health Act, local health departments, and health inspection teams that worked to address the different factors contributing to malaria. For instance, it was noted that during development of malaria control programmes at districts, several stakeholders at all government levels such as health workers, social workers and community members were involved. Another example was during the rolling out of the malaria control strategy where health workers, health educators, CHWs, health officials, health inspectors, and religious leaders were all trained which was evidence of a collective effort to addressing malaria and its social determinants.

*“When the malaria control programme was being developed, many stakeholders were brought on board, health workers, social workers, and the community. When we are rolling out the malaria control strategy, we the health workers, went for the training, health educators also went for the training, because for us as health workers, we are mostly in the facilities and the health educators are mostly in the community. The community health workers, health officials and also religious leaders were also considered, even schools.”* [KII, health facility manager]

Regular sanitation drives, such as communal cleaning days, were reported as part of these local structures and initiatives that if leveraged can ensure that breeding sites for mosquitoes were minimized. Additionally, district health teams, which oversaw malaria education and prevention activities, were reported to collaboratively work with local leaders to ensure that control measures were effectively enforced.

*“During the sanitation week or days, as sub-counties, we work with the communities to manage garbage, to clean the drainages and during those periods, the town councils or district even avail vehicles that take waste to the landfill. We clean together with the community in the campaign to ensure every place is clean and sightly.”* [KII, Wakiso district official]

CHWs were also reported to be essential structures in addressing the social determinants of malaria at community level. These were considered as trusted members of the community who conducted home visits, distributed malaria drugs and supplies, and educated communities about malaria prevention measures. It was noted that CHWs were easily accepted in communities and the majority of community members listened to them and abided by their guidelines. Participants also reported that the government and health partners had recognized the role of CHWs in addressing malaria and its social determinants, and had invested in training programmes to enhance their capacity. This was seen to improve community engagement and expand access to early diagnosis and treatment.

*“We have the community health worker programme. Community health workers come and visit us and give us malaria supplies, and we even go to their homes when our children are sick because the government gives them medicine. They have been helping us for a long time.”* [Respondent 4, FGD mixed rural]

The availability of various media channels such as community and local radios, as well as megaphones which can be used by CHWs and other district health officials to sensitise communities on malaria control strategies was seen as a great opportunity to address social determinants of malaria. It was reported that Wakiso District regularly held talk shows on various local radio stations where they talked about health issues concerning the area including malaria.

*“We have been blessed that in this village of ours, we have three local radios. Through these radios, we discuss our community perspectives on pertinent topics. On the issues of health, it is compulsory that information is disseminated and shared to the benefit of the community. Even without meeting the people physically, we do our part and share the information through the various radio programmes.”* [KII, local council leader]

## Discussion

The study revealed critical barriers and opportunities that significantly influence malaria’s persistence and control within communities. Our findings established three primary barriers to addressing the social determinants of malaria which include a predominant focus on curative interventions within national policies and private health sectors, inadequacy in coordination among key stakeholders, and a notable level of complacency at both governmental and community levels, largely originating from the endemic nature of the disease. Our study also identified two significant opportunities including the presence of a diverse range of stakeholders actively engaged in malaria control, and the existence of robust governmental structures across national, district, and community levels. These results highlight the urgent need for a paradigm shift to integrated public health strategies that effectively address the socio-economic and behavioural factors underpinning malaria transmission in Uganda. The implication of these findings is therefore highly significant for public health in the country, highlighting the necessity of championing prevention, adequate coordination, community engagement, prioritising social determinants in policies, and strategically leveraging existing structures and sectors to achieve malaria elimination.

Our study highlights a predominant focus on curative interventions as a significant barrier in Uganda’s malaria control which resonates with broader research advocating for robust preventive strategies. The findings indicates that national malaria control policies, and related government interventions in Uganda, frequently emphasize medical treatment over highly effective preventive strategies. Studies in other endemic countries such as Nigeria [26], Haiti [27], Ghana [28] and Vanuatu [29] have highlighted how crucial accessible and affordable prevention tools as well as community participation are for effective malaria control. The non-prioritization of these foundational, community-driven preventive strategies, despite their proven efficacy and cost-effectiveness, creates a significant barrier to holistically addressing the complex social determinants of malaria. This oversight consequently results into a cycle where systemic issues contributing to transmission of the disease remain unaddressed, directly impeding efforts to move beyond symptomatic treatment towards disease elimination. More emphasis on health promotion and disease prevention should be ensured, including through policy development and enforcement, resource allocation, and stakeholder engagement. This could be done not only at national level but also within district and community settings across the country.

Inadequate collaboration and coordination revealed in our study within government institutions and between partner organisations significantly limits efforts to address the social determinants of malaria. Fragmentation within the malaria control community and irregular functioning of key stakeholders creates silos that hinder a holistic response. Effectively controlling malaria, particularly in most low-resource endemic areas in Uganda, necessitates addressing broader social determinants such as housing quality, sanitation, and access to functional transport systems [30,31]. This, by its very nature, demands multi-sectoral engagement extending beyond MOH, involving crucial sectors such as public works, urban planning, and local governance [2,32]. Comparatively, similar challenges related to fragmentation and insufficient coordination have been identified and successfully mitigated in other contexts. Indeed, studies in Kenya [33] and Tanzania [34] have demonstrated the profound impact of improved inter-sectoral collaboration, enhanced capacity building, and strong community participation in addressing these issues. This has been achieved through regular multi-stakeholder meetings, facilitated discussions around a shared agenda, and emphasizing the critical need for integrated efforts. Such concerted approaches leverage the diverse strengths of various actors, move beyond isolated interventions, and enable a more comprehensive strategy for malaria control [32]. Indeed, our study findings reaffirm that when coordination is insufficient, initiatives remain disjointed, leading to substantial inefficiencies in resource allocation, duplication of efforts, and critical gaps in tackling the socio-economic factors that perpetuate malaria transmission. This fragmented approach ultimately prevents the synergistic action essential for addressing the deeply embedded social determinants that allow malaria to persist, even when various interventions are implemented. A “Health in All Policies” approach could also be explored, specifically enhancing collaboration of the Ministry of Health with other sectors such as housing, urban development, and environment. This approach could be implemented in strategic plans, guidelines, and standard operating procedures for the various sectors which have an impact on health.

The observed complacency and laxity at both community and government levels in Uganda’s malaria control efforts present another significant barrier to addressing the social determinants of the disease. Our findings indicate that this complacency largely stems from malaria’s endemic nature, leading many to perceive it as an unavoidable part of life, with a prevailing belief that little can be done to alter its course. This low-risk perception of malaria significantly undermines engagement with preventive measures and demands for an approach involving the broader social determinants of health. A study in India highlighted barriers such as lack of knowledge and reliance on informal care to malaria control [35]. However, our findings in Uganda suggest a deeper issue of behavioural normalization where a form of collective complacency hinders proactive community participation and robust governmental financial investment in preventive and social determinant-focused interventions. There is therefore a critical need to move beyond mere intervention coverage and truly address social determinants, also illustrated by recent evidence on malaria from Tanzania [36]. Their study in high malaria burden villages in Tanzania revealed over 90% bed net coverage, yet the prevalence of malaria remained high, contradicting previous findings of the country’s disease decline [36]. Therefore, the complacency in Uganda, where the endemic nature of the disease has resulted into a dangerous laxity, further exacerbates this challenge. Indeed, it prevents the sustained behavioural changes and comprehensive community engagement required to leverage interventions effectiveness. Additionally, complacency critically obstructs the recognition and strategy to address the socio-economic and environmental factors that allow malaria to persist.

The presence of multiple stakeholders, including NGOs, funding bodies, local leaders, and policy makers, emerged as a significant opportunity in our study for strengthening initiatives that address malaria’s social determinants and overall disease control efforts. This diversity of actors, each bringing unique resources, expertise, and community access, provides a robust platform that could enable a strong integrated and effective response. These findings resonate strongly with a study in Kenya that emphasized the transformative potential of collaborative frameworks [33] that move beyond traditional, siloed approaches. This allowed for comprehensive addressing of malaria’s social determinants through leveraging on shared agendas and diverse strengths [32,33]. A qualitative study in Uganda on the influence of actors in the malaria treatment policy change in Uganda also pointed out that the engagement of local leaders was instrumental in increasing community awareness of policy changes [37]. This highlighted their vital role in facilitating local stakeholder adoption and participationClick or tap here to enter text. Our study’s identification of multiple stakeholders, including local leaders, as an opportunity directly aligns with these findings. Such collaborative models, whether facilitating health education, community engagement, or resource mobilization, are critical for expanding outreach to vulnerable populations and ensuring the sustainability of interventions [37]. Strategically harnessing the power of multiple stakeholders is beneficial and essential for translating policy into actionable, as well as community-led prevention and control, thereby overcoming fragmentation of malaria interventions.

Many major funding organisations for example the Global Fund and the Gates Foundation tend not to prioritise interventions that address the broader social determinants of malaria. Their support is largely directed toward biomedical and behavioural approaches. For instance, the 2025 Global Fund Results Report highlights substantial investments in malaria control through large-scale distribution of insecticide-treated nets, indoor residual spraying, preventive treatment for pregnant women, and malaria testing and treatment [38]. Although these interventions have significantly reduced malaria cases and deaths, funding priorities remain concentrated on measurable, short-term outcomes. Limited attention is given to deeper structural and social determinants which majorly shape malaria vulnerability. This narrow focus highlights a major gap in current malaria programming and calls for more integrated strategies that combine biomedical efforts with approaches addressing the root social and environmental drivers of transmission.

Our findings also identified the existence of structures at national, district, and community levels including health inspectors, malaria focal persons, and CHWs as an opportunity for addressing the social determinants of malaria. These established frameworks provide a ready-made infrastructure for public health interventions [33] within which efforts to address social determinants of malaria could be effectively situated. CHWs are deeply embedded within communities and serve as trusted conduits for health education, distribution of supplies, and early diagnosis, directly impacting behavioural and access-related factors. Furthermore, numerous studies highlight the indispensable contribution of CHWs to malaria control [39–41]. Research from across sub-Saharan Africa has consistently demonstrated the effectiveness of CHWs in providing timely diagnosis and treatment, reducing the progression to severe malaria, and reducing treatment costs, particularly in rural areas where access to formal health facilities is limited [42,43]. The ability of CHWs to bridge the gap between formal health systems and rural communities makes them pivotal in translating policies into practical, localized interventions that address the social and environmental factors influencing malaria transmission. However, CHWs need to be adequately supported through regular training, supervision, and motivation for sustainability. Indeed, our findings indicated that the vast local stakeholders, leveraging government mandates, can address various social determinants of malaria in the local structures. This observation aligns with a study from Kenya which successfully implemented integrated vector management [33]. This emphasized the critical role of actively exploring and engaging existing community structures and networks to foster collaboration and enhance capacity. Therefore, strategically investing in and strengthening these pre-existing structures is not only cost-effective but also essential for fostering community ownership, ensuring sustained behavioural change in pursuit of malaria elimination.

A limitation of our study is having involved only one district hence the findings may not be relatable to other parts of the country. In addition, purposive sampling of participants could have introduced bias towards those who already understood malaria prevention. Use of two software in the data analysis may have introduced minor variations due to differences in data handling procedures. It is also worth noting that involving individuals in the IDIs who had lost a family member due to malaria may have caused some discomfort. However, a key strength of the study is involvement of a diverse range of stakeholders at community, sub-national and national levels hence some of the perspectives are beyond the single district involved. In addition, the use of various sources of data including FGD, KIIs, and IDIs ensured triangulation of findings. The Dahlgren-Whitehead model used in the study was also crucial in helping the participants to understand the social determinants of health. Indeed, the model facilitated discussion of important aspects related to malaria in their context. Finally, this study was designed at a general level, looking at the social determinants as a policy agenda, rather than forwarding specific policies to address particular social determinants. Whilst this is beyond scope for this article, future research is needed to understand the barriers and opportunities of implementing different interventions that address the social determinants of malaria.

## Conclusion

This study identified both barriers and opportunities influencing malaria control efforts in Uganda, through the lens of social determinants of health by considering both the proximal and distal layers of the Dahlgren-Whitehead model. The systemic barriers that prioritize short-term clinical outcomes over long-term structural prevention, emphasise persistent challenges that hinder effective disease prevention including a curative focused approach, inadequate coordination, and complacency. The opportunities, resulting from a diverse ecosystem of collaborators and established governmental structures, offer promising pathways that should be leveraged for progress. These findings underscore the need for integrated public health strategies that advocate prevention, foster robust coordination, and leverage stakeholder engagement including communities. Addressing these social determinants is not merely an adjunct to current efforts, but a fundamental requirement if Uganda is to transition from malaria control to elimination.

## Supporting information

S1 Data This is the study dataset. (DOCX)

S1 Checklist Inclusivity in global research. This is the completed questionnaire on inclusivity in global research.
(DOCX)

## References

[1] Oladipo HJ, Tajudeen YA, Oladunjoye IO, Yusuff SI, Yusuf RO, Oluwaseyi EM. Increasing challenges of malaria control in sub-Saharan Africa: priorities for public health research and policymakers. Ann Med Surg. 10.1016/j.amsu.2022.104366 PMC9421173 36046715

[2] Li J, Docile HJ, Fisher D, Pronyuk K, Zhao L. Current status of malaria control and elimination in Africa: epidemiology, diagnosis, treatment, progress and challenges. J Epidemiol Glob Health. 2024;14(3):561–79. doi: 10.1007/s44197-024-00228-2 38656731 PMC11442732

[3] Amisu BO, Okesanya OJ, Ahmed MM, Abdelbar SMM, Lucero-Prisno DE 3rd. Achieving malaria-free: Egypt’s journey to WHO certification and global implications for disease control. Trop Med Health. 2024;52(1):101. doi: 10.1186/s41182-024-00666-5 39726049 PMC11674509

[4] World Health Organization. WHO certifies Cabo Verde as malaria-free, marking a historic milestone in the fight against malaria [internet]. 2024 [cited 2026 Feb 15]. Available from: https://www.who.int/news/item/12-01-2024-who-certifies-cabo-verde-as-malaria-free--marking-a-historic-milestone-in-the-fight-against-malaria

[5] Bennett A, Smith JL. Malaria elimination: lessons from El Salvador. Am J Trop Med Hyg. 2018;99(1):1–2. doi: 10.4269/ajtmh.18-0390 29893203 PMC6085775

[6] World Health Organization. World Health Organization (2025) World Malaria Report 2024: Addressing the threat of antimicrobial drug resistance [Internet]. 2024 [cited 2026 Feb 10]. Available from: https://www.who.int/teams/global-malaria-programme/reports/world-malaria-report-2025

[7] World Health Organization. Uganda’s 24,2 hours initiative: A game changer in malaria mortality reduction [Internet]. 2025 [cited 2025 May 25]. Available from: https://www.afro.who.int/countries/uganda/news/ugandas-242-hours-initiative-game-changer-malaria-mortality-reduction

[8] World Health Organization. World Health Organization (2015) Global Technical Strategy for Malaria 2016 – 2030 [Internet]. 2015 [cited 2025 Feb 10]. Available from: https://www.who.int/docs/default-source/documents/global-technical-strategy-for-malaria-2016-2030.pdf

[9] Oladipo HJ, Tajudeen YA, Oladunjoye IO, Yusuff SI, Yusuf RO, Oluwaseyi EM, et al. Increasing challenges of malaria control in sub-Saharan Africa: priorities for public health research and policymakers. Ann Med Surg. 10.1016/j.amsu.2022.104366 PMC9421173 36046715

[10] Monroe A, Moore S, Olapeju B, Merritt AP, Okumu F. Unlocking the human factor to increase effectiveness and sustainability of malaria vector control. Malar J. 2021;20(1):404. doi: 10.1186/s12936-021-03943-4 34656116 PMC8520184

[11] Nalinya S, Musoke D, Deane K. Malaria prevention interventions beyond long-lasting insecticidal nets and indoor residual spraying in low- and middle-income countries: a scoping review. Malar J. 2022;21(1):31. doi: 10.1186/s12936-022-04052-6 35109848 PMC8812253

[12] Kooko R, Wafula ST, Orishaba P. Socioeconomic determinants of malaria prevalence among under five children in Uganda: evidence from 2018-19 Uganda Malaria Indicator Survey. J Vector Borne Dis. 2023;60(1):38–48. doi: 10.4103/0972-9062.353251 37026218

[13] Ricci F. Social implications of malaria and their relationships with poverty. Mediterr J Hematol Infect Dis. 2012;4(1):e2012048. doi: 10.4084/MJHID.2012.048 22973492 PMC3435125

[14] Nalinya S, Musoke D, Deane K. Malaria prevention interventions beyond long-lasting insecticidal nets and indoor residual spraying in low- and middle-income countries: a scoping review. Malar J. 2022;21(1):31. doi: 10.1186/s12936-022-04052-6 35109848 PMC8812253

[15] Ministry of Health. Uganda National Malaria Control Policy. Uganda. 2011 [cited 2025 Oct 8]. pp. 1–19. Available from: https://www.severemalaria.org/sites/default/files/content/document/Uganda%20NATIONAL%20MALARIA%20CONTROL%20POLICY%20-%202011%20%281%29.pdf

[16] Malaria Consortium. Uganda launches new plan to end malaria by 2030 | Malaria Consortium. Kampala, Uganda. [cited 2026 May 5]. Available from: https://www.malariaconsortium.org/news/uganda-malaria-elimination

[17] Nuwagaba E, Rutanga M. Political influence and creation of new political cities in Uganda: Challenges facing newly created cities in Uganda, the case of Mbarara City. 2025;8(9). doi: 10.36349/easjehl.2025.v08i09.001

[18] Atusingwize E, Deane K, Musoke D. Social determinants of malaria in low- and middle-income countries: a mixed-methods systematic review. Malar J. 2025;24(1):165. doi: 10.1186/s12936-025-05407-5 40420124 PMC12105309

[19] Kooko R, Wafula ST, Orishaba P. Socioeconomic determinants of malaria prevalence among under five children in Uganda: evidence from 2018-19 Uganda Malaria Indicator Survey. J Vector Borne Dis. 2023;60(1):38–48. doi: 10.4103/0972-9062.353251 37026218

[20] Semakula HM, Liang S, Mukwaya PI, Mugagga F, Swahn M, Nseka D, et al. Determinants of malaria infections among children in refugee settlements in Uganda during 2018-2019. Infect Dis Poverty. 2023;12(1):31. doi: 10.1186/s40249-023-01090-3 37032366 PMC10084630

[21] Mokuolu OA, Bolarinwa OA, Opadiran OR, Ameen HA, Dhorda M, Cheah PY, et al. A framework for stakeholder engagement in the adoption of new anti-malarial treatments in Africa: a case study of Nigeria. Malar J. 2023;22(1):185. doi: 10.1186/s12936-023-04622-2 37330469 PMC10276382

[22] Uganda Bureau of Statistics. The National Population and Housing Census 2024 – Final Report. Kampala; 2024 [cited 2025 Aug 6]. Available from: https://www.ubos.org/wp-content/uploads/2024/12/National-Population-and-Housing-Census-2024-Final-Report-Volume-1-Main.pdf

[23] Musoke D, Ndejjo R, Wafula ST, Kasasa S, Nakiyingi-Miiro J, Musoke MB. Malaria health seeking practices for children, and intermittent preventive treatment in pregnancy in Wakiso District, Uganda. Afr Health Sci. 2021;21(4):1722–32. doi: 10.4314/ahs.v21i4.28 35283976 PMC8889846

[24] Dahlgren G, Whitehead M. The Dahlgren-Whitehead model of health determinants: 30 years on and still chasing rainbows. Public Health. 2021;199:20–4. doi: 10.1016/j.puhe.2021.08.009 34534885

[25] Braun V, Clarke V. Using thematic analysis in psychology. Qual Res Psychol. 2006;3(2):77–101. doi: 10.1191/1478088706qp063oa

[26] Bamidele JO, Ntaji MI, Oladele EA, Bamimore OK. Community participation in malaria control in olorunda local government area, osun state, southwestern Nigeria. Afr J Infect Dis. 2012;6(2):24–8. doi: 10.4314/ajid.v6i2.1 23878712 PMC3578647

[27] Bardosh K, Desir L, Jean L, Yoss S, Poovey B, Nute A, et al. Evaluating a community engagement model for malaria elimination in Haiti: lessons from the community health council project (2019-2021). Malar J. 2023;22(1):47. doi: 10.1186/s12936-023-04471-z 36759860 PMC9910254

[28] Adum P, Agyare VA, Owusu-Marfo J, Agyeman YN. Knowledge, attitude and practices of malaria preventive measures among mothers with children under five years in a rural setting of Ghana. Malar J. 2023;22(1):268. doi: 10.1186/s12936-023-04702-3 37700321 PMC10498521

[29] Atkinson J-AM, Fitzgerald L, Toaliu H, Taleo G, Tynan A, Whittaker M, et al. Community participation for malaria elimination in Tafea Province, Vanuatu: Part I. Maintaining motivation for prevention practices in the context of disappearing disease. Malar J. 2010;9:93. doi: 10.1186/1475-2875-9-93 20380748 PMC2873527

[30] Cygu SB, Nabukeera B, English L, Babirye S, Gyezaho C, Ng’etich M, et al. Understanding the demographic and socioeconomic determinants of morbidity in Eastern Uganda: a retrospective analysis of the Iganga-Mayuge health and demographic surveillance data. BMJ Public Health. 2024;2(2):e000898. doi: 10.1136/bmjph-2024-000898 40018598 PMC11816860

[31] Nankabirwa JI, Gonahasa S, Katureebe A, Mutungi P, Nassali M, Kamya MR, et al. The Uganda housing modification study - association between housing characteristics and malaria burden in a moderate to high transmission setting in Uganda. Malar J. 2024;23(1):223. doi: 10.1186/s12936-024-05051-5 39080697 PMC11290271

[32] Yeka A, Gasasira A, Mpimbaza A, Achan J, Nankabirwa J, Nsobya S, et al. Malaria in Uganda: challenges to control on the long road to elimination: I. Epidemiology and current control efforts. Acta Trop. 2012;121(3):184–95. doi: 10.1016/j.actatropica.2011.03.004 21420377 PMC3156969

[33] Ng’ang’a PN, Aduogo P, Mutero CM. Strengthening community and stakeholder participation in the implementation of integrated vector management for malaria control in western Kenya: a case study. Malar J. 2021;20(1):155. doi: 10.1186/s12936-021-03692-4 33740983 PMC7977174

[34] Mlozi MRS, Rumisha SF, Mlacha T, Bwana VM, Shayo EH, Mayala BK. Challenges and opportunities for implementing an intersectoral approach in malaria control in Tanzania. Tanzan J Health Res. 2015;17(1). doi: 10.4314/thrb.v17i1.2

[35] Sundararajan R, Kalkonde Y, Gokhale C, Greenough PG, Bang A. Barriers to malaria control among marginalized tribal communities: a qualitative study. PLoS One. 2013;8(12):e81966. doi: 10.1371/journal.pone.0081966 24376507 PMC3869659

[36] Challe DP, Francis F, Seth MD, Tupa JB, Madebe RA, Mandara CI, et al. Prevalence and risk factors associated with malaria infections at a micro-geographic level in three villages of Muheza district, north-eastern Tanzania. medRxiv. 2024;2023. 10.1186/s12936-025-05506-3 PMC12487162 41035047

[37] Nabyonga-Orem J, Nanyunja M, Marchal B, Criel B, Ssengooba F. The roles and influence of actors in the uptake of evidence: the case of malaria treatment policy change in Uganda. Implement Sci. 2014;9:150. doi: 10.1186/s13012-014-0150-8 25294279 PMC4193992

[38] Global Fund. The 2025 results report. 2025 [cited 2025 Oct 8]. Available from: file:///core_2025-results_report_en.pdf

[39] Nguyen H, Jongdeepaisal M, Tuan DA, Khonputsa P, Ngo T, Pell C, et al. Sustaining village malaria worker programmes with expanded roles: Perspectives of communities, healthcare workers, policymakers, and implementers in Vietnam. PLOS Glob Public Health. 2024;4(8):e0003443. doi: 10.1371/journal.pgph.0003443 39106235 PMC11302919

[40] Adhikari B, Bayo M, Peto TJ, Callery JJ, Tripura R, Dysoley L, et al. Comparing the roles of community health workers for malaria control and elimination in Cambodia and Tanzania. BMJ Glob Health. 2023;8(12):e013593. doi: 10.1136/bmjgh-2023-013593 38070880 PMC10729139

[41] Sunguya BF, Mlunde LB, Ayer R, Jimba M. Towards eliminating malaria in high endemic countries: the roles of community health workers and related cadres and their challenges in integrated community case management for malaria: a systematic review. Malar J. 2017;16(1):10. doi: 10.1186/s12936-016-1667-x 28049486 PMC5209914

[42] Siribié M, Ajayi IO, Nsungwa-Sabiiti J, Afonne C, Balyeku A, Falade CO, et al. Training community health workers to manage uncomplicated and severe malaria: experience from 3 rural malaria-endemic areas in Sub-Saharan Africa. Clin Infect Dis. 2016;63(suppl 5):S264–9. doi: 10.1093/cid/ciw624 27941103 PMC5146696

[43] Corley AG, Thornton CP, Glass NE. The role of nurses and community health workers in confronting neglected tropical diseases in Sub-Saharan Africa: a systematic review. PLoS Negl Trop Dis. 2016;10(9):e0004914. doi: 10.1371/journal.pntd.0004914 27631980 PMC5025105

