# Supplementary material for: Barriers and opportunities to addressing social determinants of malaria in Wakiso District, Uganda
Source: PLOS Glob Public Health. 2026 Sep 9;6(9):e0006922. doi: 10.1371/journal.pgph.0006922 (PMC13557371; doi:10.1371/journal.pgph.0006922)
Supplement: Supplementary file 1 [file pgph.0006922.s001.docx]

**Social Determinants of Malaria**

Codes

Name

**Impact of Malaria**

Borrowed money for treatment

Children could not go to school

Children get jaundice

Could not work

Financial impacts

Has to close business

Hospital trips impact work time

Impact on school performance

Missed planting season

Struggled financially

**Interventions**

Burn waste

Burning cow dung as repellent

Closed businesses due to no soak pit

Communal cleaning paid for by wealthy households

Communal clearing

Community cleaning

Community clear stagnant water

Community teams address sanitation

Controls own surroundings

Cover brick making holes

Dig trenches that do not allow stagnant water

Disease surveillance

Education about sanitation

Educational programmes

Fines for poor disposal of water

Government gave drugs to VHTs

Health education around sanitation and water

Health inspectors do environmental visits

House planning

Improved house building

IRS

Keep wells clean

Larvicide

Local leadership

Net Distribution

Net Distribution at ANCs

Net provision

Not much communal work recently

Poverty acts as a barrier to community action

Provide safe water

Prune crops

Put oil in soak pits

Some communities try to improve drainage

Spray the grass

Staying indoors

Train community health workers

Use to get rid of mosquitos

Use iron roofing

Waste management

Wells cleared

Key interventions recommended by all

**Policy**

Advice for farmers

Advocacy and social mobilisation

Budget issues

Budgets have not kept pace with population

Budgets not used well

Building standards

Can't intervene in people’s homes

Challenges matching plan with funding

Challenges with urban slums

Communities not involved

Corruption

Current strategies not enough

Delays in local laws

Differences in central government and local leaders’ views

Different settings

Donors downplay local solutions

External funding

Fear of upsetting voters

Finance for health infrastructure

Finance is a barrier

Focus on behaviour and the poor as lazy

Focus on behaviour change

Focus on treatment

Good governance in the health system

Government budget spent on other things

Government can't afford enough health workers

Government does not engage with traditional healers

Government doesn't distribute enough nets

Government focuses on key diseases

Government focusing on treatment

Government has a people centred approach

Government has ensured health facilities are stocked

Government has invested in training

Government health facilities have free medicine

Government needs to invest in health

Government spends a lot on malaria

Health officials are no longer there

Health officials no longer supervise waste

Households need to implement interventions

Ignorance as barrier to addressing SDOH

Implementation is challenging

Implementation is politicised

Inequality

Interventions need to be free

Lack of public health officers

Laws not being enforced

Limited power to deal with neighbours

Malaria has been politicised

Malaria needs to be addressed in the community

Malaria needs to be mainstreamed

Malaria work skewed towards clinical side

Ministers not aware about local challenges

Multisectoral approach

Myths about political motivations

Need continued support

Need evidence for impact of SDOH

Need good leadership

Need insecticides

Need more health facilities

Need more research on SDOH

Need quality housing

Need to bring in all stakeholders

Need to focus on households

Need to focus on prevention

Need to integrate SDOH

Need to spray everywhere

Need to strengthen health system

Need to work as one

Nets and medicine are not enough

Nets not enough

NGOs need to integrate

No funding for SDOH

No partners address SDOH

No programmes expect net distribution

Not easy to approach people about their plot

Not enough nets per household

Not much change in strategy

Only receive nets

People stopped having sanitation days

Policies do not address SDOH

Policies do not address working conditions

Politicians slowing down programmes

Poverty as a barrier to action

Prevention is politicised

Projects should be bottom up

Reliance on donors for funding

Resources diverted

SDOH not addressed in development strategies

Short term programmes

Smart homes

Strategies written well

Supply chain issues

Support with access to treatment

Tenants have no rights

Too much focus on nets

VHTs are good

Won’t use interventions from other political parties

Working in Silos

**Suggested Interventions**

Be indoors by 7pm

Bring back public health officers

Bring cows back before 6pm

Clean up animal urine

Clear bushes

Community garbage collection

Cover over drainage channels

Encourage ventilators in housing

Enforce laws about urban agriculture

Ensuring a clean environment

Find out where mosquitos sleep

Fine people who dump garbage

Free garbage collection

Free treatment

Getting the cows from the bush

Give testing kits to VHTs

Government improve houses

Grandparents used to smoke homes

Health education

Identify trees to plant

Improve dealing with waste

Improve water supply

Improved clothing for work

Improved drug supply

Increase supply of drugs

Invest in VHTs

Involve all stakeholders

Laws about clearing land

More communal work

More funding for ministry of health

More VHTs

Need to co-operate

Pay unemployed youth to clean areas

Penalise net misuse

Piped water supply

Plant crops far from home

Plant grass that repels mosquitos

Plant Neem tree

Pour oil in holes

Rear fish to ear the larvae

Spray crops

Spray swamps

Spraying everywhere prevents crop exports

Stop school study at night

Tell neighbours to drain stagnant water

Train village chairpersons

VHTs need more resources

**The Social Determinants**

**Agriculture**

Agricultural activities

Agriculture beans leaves keep water

Coffee plantations

Food security and malaria

Go to gardens early in the morning

Growing Bananas

Growing beans

Growing maize around households

Growing potatoes

Growing yam in the swamp

Long days in agricultural sector

Multiple use of chemicals creating resistance in mosquitos

Plantations

Planting season

Rice cultivation

Stagnant water due to agriculture

Urban agriculture

Urine is a breeding place

Water storage of agriculture

**Animal Husbandry**

Animal husbandry practices

Animals around homes

Farming fish in ponds

Fetching cows in the evening

Guarding cattle

Keeping cattle indoors

Keeping cows, pigs and goats

Keeping goats in shelters

Milk cows in evening and early morning

Pond for ducks

Poor hygiene practices

Bitten before bed

Bitten during the day

Bitten outside the household

Community level impacts

Cross border travelling after market

Difference between poor and non-poor

Different environments

**Education**

Education level is important

Ignorance is the main issue

Inequalities in education

Low levels of education are an issue

Men ignore malaria messages

**Environment around homes**

Building in wetlands

Bushes around homes

Bushy areas

Can’t afford to but dry land

Food stores

High population density in the community

Live near forests

Live near lake

Living near landing sites

Living near swamps and water

Makeshift fences

Not using toilets

Poor hygiene around houses

Poor live nearer breeding sites

Rents house in waterlogged environments

Vacant land

Waste around homes

Going to school early

**Health system**

Long wait times at health facilities

Lots of private health facilities

Men make decisions about access to treatment

Need to buy medicine

Poor quality health services

Prompt diagnosis

Stock outs of medicine

**Housing**

Bathrooms have uncovered holes

Can't afford good housing

Cheap housing

Congested housing

Good housing quality

Grass roofing

High population density

Homes do not have ventilators

Houses are taxed

Houses do not have soak pits

Houses with ventilators

Housing is expensive

Housing mosquito bites in sitting room

Housing with no windows

Lack of planning for new buildings

Latrine systems

Living in not completed houses

No building plans

People sleep outside

Pit latrines

Poor housing quality

Semi-permanent housing

Unsuitable doors

Urban poverty and poor housing

Land shortages and smaller plots

**Livelihoods**

Bar workers

Building sites

Challenge with evening workers

Cooking food

Engaging in sex work at night

Evening workers

Fetching firewood

Fishing at night

Health workers don't ask about work conditions

Hotel workers

Lack of follow up due to livelihood activities

Looking for work at night

Market vendors

Pump attendant

Road construction

Rubbish in the market

Sand mining

Security work

Sell chips by the road

Selling food and agricultural products

Truck driving

Truck driving (2)

Waiting for customers

Work early in the morning

Work in forests

Work near drainage channels

Work near swamps

Work on brick laying sites

Workers unloading at night

Working late at night

Works on the lake

Mosquitoes move

**Poverty related issues**

Can't afford nets

Can't afford repellents

Can't afford to repair housing

Can't afford treatment

Can't afford waste collection

Can't afford whole dose

Can't buy a hoe

Grow maize as need money

Lack of food when taking treatment

Malaria is associated with poverty

Malaria rates and poverty

Malnourishment

Nets used to protect crops

Poor don't have time for talks about malaria

Poor lose hope

Poverty

Poverty as a barrier to health seeking

Resort to traditional methods

Sell nets

Think about food not health

Treatment is expensive

Weakened immune system

Repellents

School students study in evenings

Schools near swamps

Seasonal chemoprevention

**Social events**

Church attendance at night

Funerals

Weddings

Worship

**Traditional methods**

Traditional beliefs

Traditional beliefs as a barrier to addressing malaria

Traditional healers

Use local herbs

Witchcraft

Travelling to endemic areas

**Water and Sanitation**

Bite children when they go to collect water

Boreholes better than wells

Collecting water at the lake

Collecting water at the well

Community action doesn't happen anymore

Drainage channels full of waste

Drainage channels not directed well

Drainage channels

Fetching water from open wells

Lots of water

Need piped water

Pits for brick making

Ponds as a breeding ground

Poor sanitation

Poor sanitation in urban areas

Potholes

Puddles

Rainy seasons

Saving water in buckets and open tanks

Slums and stagnant water

Soak pits have stagnant water

Stagnant water

Stagnant water in densely populated areas

Waste and rubbish sites

Waste in drainage channels

Water needs to flow

Water storage

Weather conditions

Wealthy can afford to protect themselves

Wealthy take prophylaxis

Views on social determinants

Africans have higher immunity

Financing focused on nets and IRS

Focus on nets

Good this research is being done

Malaria as a community disease

Malaria is a serious issue

Malaria is common

Malaria is normal

Malaria not a priority

Malaria still an issue

Need to account for social and cultural context

Need to prioritise prevention

Nets and IRS not enough

No effort to address SDOH

Poverty is a barrier to addressing SDOH

Seeing more cases in adolescents

Social determinants not addressed

Social determinants not well understood

Water and Sanitation not addressed
